# Enhancing Pharmacy Student Learning and Perceptions of Medical Apps

**DOI:** 10.2196/mhealth.4843

**Published:** 2016-05-12

**Authors:** Jennifer Rodis, Timothy Dy Aungst, Nicole V Brown, Yan Cui, Leonard Tam

**Affiliations:** ^1^ The Ohio State University College of Pharmacy Division of Pharmacy Practice and Science Columbus, OH United States; ^2^ MCPHS School of Pharmacy Worcester, MA United States; ^3^ The Ohio State University Center for Biostatistics Columbus, OH United States; ^4^ The Ohio State University College of Pharmacy Columbus, OH United States

**Keywords:** mobile applications, pharmacy, students, health care

## Abstract

**Background:**

The use of mobile apps in health care is growing. Current and future practitioners must be equipped with the skills to navigate and utilize apps in patient care, yet few strategies exist for training health care professional students on the usage of apps.

**Objective:**

To characterize first-year pharmacy student use of medical apps, evaluate first-year pharmacy student's perception of skills in finding, evaluating, and using medical apps before and after a focused learning experience, and assess student satisfaction and areas for improvement regarding the learning experience.

**Methods:**

Students listened to a recorded, Web-based lecture on finding, evaluating, and using mobile apps in patient care. A 2-hour, interactive workshop was conducted during which students were led by an instructor through a discussion on strategies for finding and using apps in health care. The students practiced evaluating 6 different health care–related apps. Surveys were conducted before and after the focused learning experience to assess students' perceptions of medical apps and current use and perspectives on satisfaction with the learning experience and role of technology in health care.

**Results:**

This educational intervention is the first described formal, interactive method to educate student pharmacists on medical apps. With a 99% response rate, surveys conducted before and after the learning experience displayed perceived improvement in student skills related to finding (52/119, 44% before vs 114/120, 95% after), evaluating (18/119, 15% before vs 112/120, 93% after), and using medical apps in patient care (31/119, 26% before vs 108/120, 90% after) and the health sciences classroom (38/119, 32% before vs 104/120, 87% after). Students described satisfaction with the educational experience and agreed that it should be repeated in subsequent years (89/120, 74% agreed or strongly agreed). Most students surveyed possessed portable electronic devices (107/119, 90% mobile phone) and agreed with the concept of medical apps being an important part of the health care profession in the future (112/119, 94% before and 115/120, 96% after).

**Conclusions:**

Student pharmacists recognize the key role technology plays in the future of health care. A medical apps workshop was successful in improving student pharmacists' perceptions of ability to find, evaluate, and use medical apps.

## Introduction

With the worldwide popularity of mobile devices (eg, mobile phones, tablet computers), mobile apps are increasingly being used by health care professionals in a variety of settings. A mobile device is defined as “a portable, wireless computing device that is small enough to be used while held in the hand” [1]. In the health care field, it is estimated that there will be 500 million smartphone users worldwide with mobile health apps by 2015, and the global market for these apps may reach US $26 billion by 2017 [2]. There are more than 43,000 health care, fitness, and medical apps available in English on iTunes [3]. As mobile technology continues to gain popularity, health care practitioners, students, and residents are gravitating toward the utilization of mobile devices to assist in their clinical duties and education [4-9].

Clinicians have access to mobile medical apps that serve as drug references, clinical calculators, disease references, and clinical decision-processing aides [10,11]. Pharmacists have also integrated mobile devices as a means to help process medical orders in the hospital, access clinical references, and increase communication with providers in their practices and professional duties [12-14]. Although medical apps are helping advance the field of health care in this digital era, there are certain pitfalls being noted by the health care and academic communities related to the quality of the apps and evidence of health information provided in them [15-17]. One of the biggest issues is the reliability and accuracy of information found within the plethora of medical apps available. Several studies have identified these concerns, with issues ranging from lack of medical evidence, nondisclosure of authorship, design flaws, and inaccurate information [18-21].

The Food and Drug Administration (FDA) has recently released guidelines on which subset of medical apps it will review and regulate [22]. Whereas the safety of such apps is being ensured by FDA standards, apps that record life events, extract medical content, serve as clinical or drug references, or facilitate the communication between clinicians or health centers with patients will not be regulated by the FDA [17]. Given the limited scope of FDA in monitoring medical apps, relevance and reliability of these apps will need to be determined by the users. As health care professionals, pharmacists should have the adequate background and understanding of these apps to critique the information provided, to be able to recommend to patients or colleagues, and to use them effectively when providing care.

Future health care practitioners will likely be the generation to formally adopt mobile technology into their workflow and clinical practice. Studies have identified that mobile devices and medical apps are increasingly being incorporated into formal medical education [23-25]. However, strategies on educating current and future pharmacists to use apps effectively have not been reported. The objectives of this study were to characterize first-year pharmacy student use of medical apps, evaluate first-year pharmacy student's perception of skills in finding, evaluating, and using medical apps before and after a focused learning experience, and assess student satisfaction and areas for improvement regarding the learning experience.

## Methods

### Description of Learning Experience

Faculty from The Ohio State University College of Pharmacy (OSU COP) and Massachusetts College of Pharmacy and Health Sciences (MCPHS) University School of Pharmacy collaborated to create a novel learning experience for pharmacy students that focused on finding, evaluating, and using medical apps. The project was approved as exempt research by The Ohio State University Institutional Review Board. At OSU COP, foundational concepts related to drug information, including literature assessment, are taught in the spring semester of the PharmD curriculum as a distinct module that is part of an introductory pharmacy practice course series. During this module, students are engaged with didactic, workshop, and project-based experiences focused on finding, evaluating, and using drug information in patient care. This medical app educational experience was integrated into this course. This two-part learning experience involved a prerecorded, Web-based lecture that students were required to view before arriving for a 2-hour, small group workshop of approximately 30 students to apply concepts learned in the lecture. Available for 1 week before the workshop, the 38-minute lecture was created by the faculty member from MCPHS, video recorded via QuickTime, and uploaded onto a private view YouTube profile made available to OSU COP students. The lecture content was based on the role of mobile medical apps in clinical practice, issues and opportunities with utilization of medical apps, and how to review and assess medical apps. The lecture demonstrated to students how to evaluate mobile medical apps with several examples provided. In addition to the lecture, a tool for evaluating medical apps was shared with students; they were required to bring a copy of this tool with them to the workshop. This tool was developed by one of the coinvestigators and previously published incorporating previous strategies published [26,27].

Prior to the workshop, students were sent a list of medical apps that could be used free of charge and were available for download to portable electronic devices with iOS or Android operating systems, such as mobile phones or tablets. Students were asked to download these apps to a device, if available, before the workshop. Medical apps were selected by faculty and then reviewed before the start of the workshop. Identification of apps was conducted via previously identified means [26], with an emphasis on apps related to pharmacy practice, drug information, medical calculators, and general clinical knowledge. Apps were reviewed for both positive and negative qualities to spur discussion among students regarding evaluation techniques during the course of the workshop. The medical apps selected for inclusion in the workshop are detailed in Table 1.

**Table 1:** Mobile apps used in class activity.

Mobile app	Operating system
Medscape	iOS and Android
BodyXQ Heart	iOS and Android
Glucagon	iOS
Psych Drugs	iOS and Android
Cardiology Tool by Epocrates	iOS
Managing Dabigatran	iOS and Android

When students arrived to class, a discussion was led by the instructor to review key concepts covered in the previously posted Web-based lecture, including how to use the medical app evaluation tool. The discussion also involved a facilitated conversation about where students find apps and students' experience with apps that had been useful or not useful. Students were split into groups of 4 to 5 students each (6 groups in total per workshop) to evaluate up to 6 medical apps. Each group presented their experience using the evaluation tool to the class and discussed what the individual groups had found with a focus on challenges in evaluating medical apps as well as how to determine what apps are useful in practice.

### Evaluation of Learning Experience

Metrics were collected from YouTube Analytics on the number of views and visits received before the start of the workshop. To evaluate the effect of these medical apps on learning experience, prospective surveys were conducted before and after the involvement of first-year pharmacy students in the learning experience (see Multimedia Appendices 1 and ). The surveys were created by the faculty collaborators on the project from OSU COP and MCPHS to assess changes in students' perceptions regarding how to find, evaluate, and use medical apps in pharmacy. Descriptive questions were asked to characterize the use of portable electronic devices and medical and nonmedical apps by this student population. The first survey included 27 questions involving 5-point Likert scale format (strongly agree to strongly disagree) for perceptions on student confidence with finding, evaluating, and using medical apps. Multiple choice, check all that apply, and open-ended questions were also included to gather information regarding app and device use. The second survey included 18 questions involving the same 5-point Likert scale format questions as well as many of the same multiple choice and check-all-that-apply questions for comparison with the first survey's responses. Demographic data collection about device ownership and population characteristics was not repeated. The second survey additionally asked about satisfaction with the learning experience. Deidentified data were entered into an Excel spreadsheet, then analyzed descriptively in aggregate with summary statistics, including 95% confidence intervals for proportions where appropriate, which were generated in SAS v9.2 (SAS Institute, Cary, NC).

Both surveys were conducted during the workshop as paper surveys. The first survey was conducted on the first day of workshop for the semester. The second survey was administered during the workshop 2 weeks after the medical apps workshop experience.

## Results

### Pharmacy Student Use of Mobile Applications

The first-year class includes a total of 120 students. There were 96 visits to the YouTube video before the start of the workshop. From the first-year pharmacy class of 120 students, 119 students completed the first survey whereas 120 students completed the second survey. Differences in response total were due to 1 student being absent from class the day the first survey was conducted. Demographics of students gathered are shown in Table 2 and are typical of a first-year PharmD class across the United States. Of 119 students, 107 (90%) own a mobile phone and 86 (72%) own a portable electronic device.

**Table 2:** Demographics (N=119).

Characteristic		n (%)
Sex, female		75 (63)
Age, years			
	18-25	100 (85)	
	25-30	15 (13)	
	31-35	2 (2)	
	36-40	1 (1)	
Race			
	Caucasian	90 (77)	
	African American or black	4 (3)	
	Asian	21 (18)	
	Other	2 (2)	
Device ownership		114 (96)

On the basis of the results of the first survey, most students learned about apps via word of mouth, including obtaining information from classmates (96/119, 81%), social media such as Facebook, Twitter, and blogs (58/119, 49%), pharmacy staff at work (44/119, 37%), and family (44/119, 37%; Table 3).

**Table 3:** Responses regarding where students learn about new apps, before and after the learning experience.

Source	Before (n=119)			After (n=120)		
	n	%	95% CI	n	%	95% CI
Family	44	37	28-46	22	18	11-25
Friends or classmates in pharmacy school	96	81	74-88	100	83	77-90
Friends or classmates from other health professions schools	27	23	15-30	33	27	20-35
Medical or pharmacy Staff where I work	44	37	28-46	57	47	39-56
Facebook	38	32	24-40	19	16	9-22
Twitter	11	9	4-14	2	2	0-4
Blogs	9	8	3-12	6	5	1-9
News	26	22	14-29	14	12	6-17
Professional organizations	11	9	4-14	8	7	2-11
Other	4	3	0-7	12	10	5-15
None of the above or N/A^a^	7	6	2-10	6	5	1-9

^a^N/A: not applicable.

**Figure 1:**
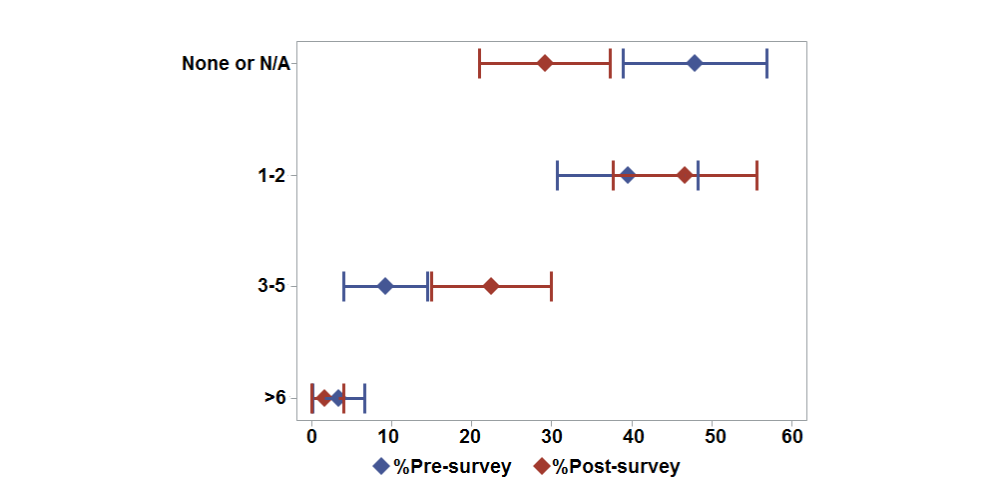
Medical apps owned per student. Proportions (diamonds) and 95% confidence intervals (horizontal lines) for the surveys conducted before (pre-survey) and after (post-survey) the learning experience. N/A: not applicable.

In addition, most students reported regularly using 0 to 2 medical or pharmacy-related apps and approximately 55% (65/119) of students indicated currently having a drug information app installed on a portable electronic device (Figure 1).

### Impact of Learning Experience on Student Perceptions of Mobile Applications

**Figure 2:**
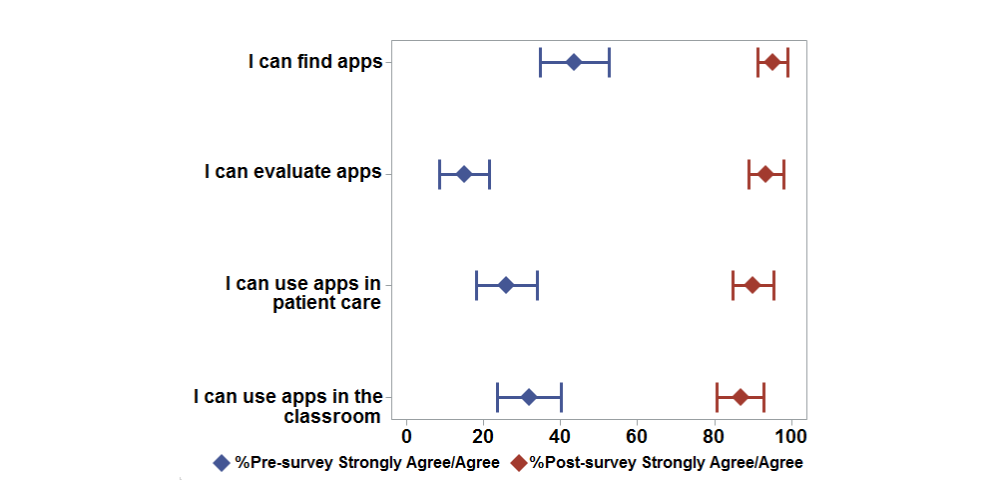
Student assessment of evaluation technique. Proportions (diamonds) and 95% confidence intervals (horizontal lines) for the surveys conducted before (pre-survey) and after (post-survey) the learning experience.

Before the workshop, most students indicated that medical or pharmacy-related apps are beneficial to pharmacy practice (98/119, 82%) and that mobile technology will influence pharmacy practice in the future (112/119, 94%). However, less than 44% of students agreed or strongly agreed that they knew how to find (52/119, 44%), evaluate (18/119, 15%), or use medical or pharmacy-related apps (31/119, 26%; Figure 2).

The students cited lack of knowledge of apps and inability to recognize when it was appropriate to use a mobile device in practice as the two main barriers to using mobile devices in pharmacy practice (Table 4).

**Table 4:** Responses regarding student-identified barriers to using apps, before and after the learning experience.

Barriers to using apps	Before (n=119)			After (n=120)		
	n	%	95% CI	n	%	95% CI
Lack of knowledge of apps	87	73	65-81	69	58	49-66
Technical difficulty	37	31	23-39	37	31	23-39
Purchasing a device	35	29	21-38	49	41	32-50
Recognizing when it's appropriate to use one	66	55	47-64	66	55	46-64

The second survey's responses indicated changes in students' perceptions in a variety of areas after participating in the medical apps learning experience (Figure 2). After the workshop, more than 90% of students agreed or strongly agreed that they knew how to find (114/120, 95%), evaluate (112/120, 93%), and use medical apps (108/120, 90%), compared with 43% before the workshop (Figure 2). Most students also reported increased usage of apps than on the first survey with up to 5 medical or pharmacy-related apps regularly used (Figure 1). Eighty percent of students (96/120) indicated that a drug information app was currently installed on one of their mobile devices. The source of information about new apps also changed after the workshop, with students indicating that they relied more on colleagues than social media and family to learn about new apps (Table 3). Perceptions on benefits and barriers to using mobile devices in pharmacy changed minimally, with the percentage of students agreeing or strongly agreeing that apps benefit pharmacy practice and that mobile technology will influence pharmacy practice increasing from 95% (113/119) to 96% (115/120). Lack of knowledge was indicated as a barrier less often in the second survey compared with the first survey (Table 4). Also, students' perceptions related to the cost of a medical or pharmacy-related app did not differ between first and second survey data. Most students were willing to pay less than US $2.99 for a medical or pharmacy-related app. Overall, most students found the medical apps presentation and the activities to be useful (at least 70% agreed or strongly agreed, 84/120) and that they would suggest offering the activity in the class next year (74% agreed or strongly agreed, 89/120).

## Discussion

This educational intervention is the first described formal, interactive method to educate pharmacy students on medical apps. Comparative surveys conducted before and after the experience display a perceived improvement in student skills related to finding, evaluating, and using medical apps. Students described satisfaction with the educational experience and agreed that this experience should be repeated in subsequent years. Most students surveyed possessed portable electronic devices, used apps regularly, and agreed with the concept of medical apps being an important part of the health care profession in the future.

Current literature reveals that medical trainees in a variety of practice environments share similar trends and perceptions of medical apps. A study of US urology trainees found that 77% reported downloading apps with 30.6% also paying for them; the mean number of apps downloaded was 4 (range 1-12). Approximately 44% of trainees indicated apps for mobile phones as being very useful in clinical practice [28]. Our pharmacy students reported similar app-downloading habits, with a much higher percentage believing apps are integral to the practice. Methods to evaluate apps have been published, including the tool used in this project [26,27,29]. No models for educating health care professionals on finding, evaluating, or using apps have been described. Greater emphasis on educating future health care practitioners in the classroom on the appropriate use of mobile technology has been suggested [30].

Although our study demonstrated that students felt more comfortable with evaluating mobile medical apps, our results indicated that they still felt they would benefit from greater knowledge on when it is appropriate to use the technology in practice. Although this was not a focus of our study, our results suggest possible benefit from addressing the topic of e-professionalism and the integration of mobile devices into practice in pharmacy and possibly other health sciences curricula.

Limitations of this project relate to the narrow scope of the educational intervention. This intervention occurred in one course at one pharmacy school with first-year pharmacy students who have not yet experienced patient care at the level of more advanced students and may not be ready to apply the experience to real-life medication management in practice. Although the faculty attempted to choose a variety of medical apps to evaluate in one workshop session, we were able to accommodate up to 6 total apps. A greater variety may have allowed for a more in-depth discussion through application to core concepts. Approximately 80% of the class viewed the preparatory Web-based training module before the workshop, though it cannot be determined if each video view was conducted by each student alone or in groups. Despite this, data were analyzed in aggregate and showed overall improved perceptions by the majority of the class. Another limitation to considering the effect of this experience is that faculty evaluated student perception of skills with no formal assessment of student abilities. Data on students' general use of digital technology, including social media, were evaluated as demographic information to describe the population and not assessed in the second survey; thus, investigators are unable to identify any effects this learning experience may have had on this digital technology usage. Additionally, unique identifiers were not collected to match each student's responses for the first and second data collection. Consequently, no formal hypothesis testing for the changes before and after the learning experience could be performed and analyses of the data were limited to descriptive summaries.

Medical apps will be an inevitable component of health care in the 21st century. Pharmacy and other health care professionals must be equipped with the skills to navigate this new open-access world to provide safe and effective recommendations and care of patients. An important element of the portable technology world is medical apps. This paper describes a model for engaging pharmacy students in an active learning experience in finding, evaluating, and using medical apps. This model is transferable to other colleges of pharmacy as well as other health care professional training programs aimed at both current and future practitioners.

## Appendix

Multimedia Appendix 1 Pre-Survey.

Multimedia Appendix 2 Post-Survey.

## Abbreviations

FDA: Food and Drug Administration
MCPHS: Massachusetts College of Pharmacy and Health Sciences
OSU COP: The Ohio State University College of Pharmacy
