# Supplementary material for: Enhancing Pharmacy Student Learning and Perceptions of Medical Apps
Source: JMIR Mhealth Uhealth. 2016 May 12;4(2):e55. doi: 10.2196/mhealth.4843 (PMC4882412; doi:10.2196/mhealth.4843)
Supplement: Multimedia Appendix 1 [file mhealth_v4i2e55_app1.pdf]

Medical Apps Survey 1 ___ **P1** ___ **P2** ___ **P3**

Please indicate your agreement with the following statements on a 5 point scale:	1= Strongly Disagree	2 = Disagree	3 = Neutral	4 = Agree	5 = Strongly Agree
I know how to find medical or pharmacy apps that are useful to me					
I know how to evaluate medical or pharmacy apps					
I know how to use medical or pharmacy apps to enhance the care I provide to patients in pharmacy practice learning environments (i.e., experiential, internship)					
I know how to use medical or pharmacy apps to help me study or complete classroom-based assignments					
Using a mobile device improves my efficiency					
Medical or pharmacy apps are beneficial to pharmacy practice					
Mobile technology will influence pharmacy practice in the future					
Mobile technology should be integrated into pharmacy curricula					

Do you own a smartphone?
___ Yes ___ No

What type of smartphone do you have?
___ Android Phone (e.g. Samsung Galaxy, Nexus 5)
___ iPhone
___ Blackberry Phone
___ Windows Phone (e.g. Lumia, HTC 8X)
___ I do not own a smartphone
___ Other (specify) ____

Do you own any portable electronic devices (e.g. tablet computer, iPad)?
___ Yes ___ No

Please indicate all other portable electronic device(s) you use regularly.

___ iPad
___ iPad Mini
___ iPod
___ Surface RT/Pro vice
___ Kindle
___ Nook

___ Android based Tablet (e.g. Nexus 7)
___ Palm
___ I do not own a portable electronic device
___ Other (please list):_______________

How often do you use your mobile device:	Never	Daily	Weekly	Monthly	Quarterly
As a Calculator?					
To play games?					
To place a phone call?					
To text?					
To explore social media?					
For scheduling?					

How many total non-medical/pharmacy apps do you currently have installed on any portable electronic device(s) (including smartphones) you use regularly?

___ None or N/A
___ 1-2
___ 3-5
___ 6-8
___ 9-10
___ more than 10

How many total medical or pharmacy apps do you currently have installed on any portable electronic device(s) (including smartphones) you use regularly?

___ None or N/A
___ 1-2
___ 3-5
___ 6-8
___ 9-10
___ more than 10

What type of medical of pharmacy apps to you download? (check all that apply)

___ Drug information
___ Medical Calculators
___ News
___ Journals
___ e-Books
___ Clinical references
___ Other: _________________________

I learn about new apps from (check all that apply):
__Family
__Friends/Classmates in Pharmacy School
__Friends/Classmates from other Health Professions School(s)
__Medical/Pharmacy Staff where I work
__Facebook
__Twitter
__ Blogs
__ News
__Professional organizations (specify) ____________
__Other, specify____________
__None of the above or N/A

Which of the following are barriers to using mobile devices in pharmacy (Check all that apply)?
__ Lack of knowledge of apps
__ Technical difficulty
__ Purchasing a device
__ Recognizing when it is appropriate to use a mobile device
__ Other: ____

How much would you be willing to pay for a medical or pharmacy-related app?
___ $0.00 ___ $0.99 ___ <$2.99 ___ <$4.99 ___ <$9.99

What is your Gender?
___ Male ___ Female

What is your Age (in years)?
___ 18-25 ___ 25-30 ___ 31-35 ___ 36-40 ___ 41-45 ___ more than 45 years

What is your Race?
___ Caucasian ___ Hispanic ___ African American/Black ___ Asian ___ Other
