# Supplementary material for: Enhancing Pharmacy Student Learning and Perceptions of Medical Apps
Source: JMIR Mhealth Uhealth. 2016 May 12;4(2):e55. doi: 10.2196/mhealth.4843 (PMC4882412; doi:10.2196/mhealth.4843)
Supplement: Multimedia Appendix 2 [file mhealth_v4i2e55_app2.pdf]

**Medical Apps Survey 2**

| Please indicate your agreement with the following statements on a 5 point scale: | 1= Strongly Disagree | 2 = Disagree | 3 = Neutral | 4 = Agree | 5 = Strongly Agree |
|---|---|---|---|---|---|
| I know how to find medical or pharmacy apps that are useful to me | | | | | |
| I know how to evaluate medical or pharmacy apps | | | | | |
| I know how to use medical or pharmacy apps to enhance the care I provide to patients in pharmacy practice learning environments (i.e., experiential, internship) | | | | | |
| I know how to use medical or pharmacy apps to help me study or complete classroom-based assignments | | | | | |
| Using a mobile device improves my efficiency | | | | | |
| Medical or pharmacy apps are beneficial to pharmacy practice | | | | | |
| Mobile technology will influence pharmacy practice in the future | | | | | |
| Mobile technology should be integrated into pharmacy curricula | | | | | |

How many total non-medical/pharmacy apps do you currently have installed on any portable electronic device(s) (including smartphones) you use regularly?

___ None or N/A
___ 1-2
___ 3-5
___ 6-8
___ 9-10
___ more than 10

How many total medical or pharmacy apps do you currently have installed on any portable electronic device(s) (including smartphones) you use regularly?

___ None or N/A
___ 1-2
___ 3-5
___ 6-8
___ 9-10
___ more than 10

What type of medical of pharmacy apps to you download? (check all that apply)

___ Drug information
___ Medical Calculators
___ News
___ Journals
___ e-Books
___ Clinical references
___ Other: ______________________________________________

I learn about new apps from (check all that apply):
__Family
__Friends/Classmates in Pharmacy School
__Friends/Classmates from other Health Professions School(s)
__Medical/Pharmacy Staff where I work
__Facebook
__Twitter
__ Blogs
__ News
__Professional organizations (specify) _____________
__Other, specify_____________
__None of the above or N/A

Which of the following are barriers to using mobile devices in pharmacy (Check all that apply)?
__ Lack of knowledge of apps
__ Technical difficulty
__ Purchasing a device
__ Recognizing when it is appropriate to use a mobile device
__ Other: _________________________________________________________________

How much would you be willing to pay for a medical or pharmacy-related app?
___ $0.00 ___ $0.99 ___ <$2.99 ___ <$4.99 ___ <$9.99

The following table asks questions pertaining to the medical apps recitation.

| Please indicate your agreement with the following statements on a 5 point scale: | 1= Strongly Disagree | 2 = Disagree | 3 = Neutral | 4 = Agree | 5 = Strongly Agree |
|---|---|---|---|---|---|
| The medical apps presentation was useful. | | | | | |
| The activities in recitation were useful. | | | | | |
| I would suggest offering this activity in this class next year. | | | | | |

Please include suggestions for improvement to the activity in the space provided below:
